# The evolutionary history and global spatio-temporal dynamics of potato virus Y

**DOI:** 10.1093/ve/veaa056

**Published:** 2020-11-21

**Authors:** Fangluan Gao, Shusuke Kawakubo, Simon Y W Ho, Kazusato Ohshima

**Affiliations:** Institute of Plant Virology, Fujian Agriculture and Forestry University, Fuzhou 350002, China; Laboratory of Plant Virology, Department of Biological Sciences, Faculty of Agriculture, Saga University, 1-banchi, Honjo-machi, Saga 840-8502, Japan; School of Life and Environmental Sciences, University of Sydney, Sydney, NSW 2006, Australia; Laboratory of Plant Virology, Department of Biological Sciences, Faculty of Agriculture, Saga University, 1-banchi, Honjo-machi, Saga 840-8502, Japan; The United Graduate School of Agricultural Sciences, Kagoshima University, 1-21-24 Korimoto, Kagoshima 890-0065, Japan

**Keywords:** potato virus Y, Bayesian phylogenetics, viral phylodynamics, phylogeography, time-structured sequence data, substitution rate

## Abstract

Potato virus Y (PVY) is a destructive plant pathogen that causes considerable losses to global potato and tobacco production. Although the molecular structure of PVY is well characterized, the evolutionary and global transmission dynamics of this virus remain poorly understood. We investigated the phylodynamics of the virus by analysing 253 nucleotide sequences of the genes encoding the third protein (P3), cylindrical inclusion protein (CI), and the nuclear inclusion protein (NIb). Our Bayesian phylogenetic analyses showed that the mean substitution rates of different regions of the genome ranged from 8.50 × 10^−5^ to 1.34 × 10^−4^ substitutions/site/year, whereas the time to the most recent common ancestor of PVY varied with the length of the genomic regions and with the number of viral isolates being analysed. Our phylogeographic analysis showed that the PVY population originated in South America and was introduced into Europe in the 19th century, from where it spread around the globe. The migration pathways of PVY correlate well with the trade routes of potato tubers, suggesting that the global spread of PVY is associated with human activities.

## 1. Introduction

Owing to their high mutation rates, short replication cycles, and large population sizes, RNA viruses exhibit extreme evolutionary dynamics (Garcia-Arenal, Fraile, and Malpica 2001). The evolutionary rates and timescales of these viruses can often be inferred from phylogenetic analysis of sequence data sampled at different points in time, because genetic change accumulates in these viruses even over short epidemiological timescales (Duchene et al. 2016). Bayesian phylodynamic inference is one of the most widely used approaches for estimating the origin of epidemics and tracking the geographic spread of pathogens (Pybus and Rambaut 2009). Although this method is frequently used in studies of influenza viruses and other rapidly evolving pathogens of animals (Murcia, Wood, and Holmes 2011; Bahl et al. 2013; Su et al. 2015; Trovão et al. 2015; Magee, Suchard, and Scotch 2017), it has rarely been applied to plant viruses except where there has been a focus on inferring the evolutionary timescale (Yasaka et al. 2015, 2017; Duan et al. 2018; Gao et al. 2019). Understanding the evolution of emerging plant viruses can inform management practices for plant diseases, leading to benefits for researchers and farmers in combating such diseases in crops (Roossinck 2008).

*Potato virus Y* (PVY), the type species of the genus *Potyvirus* from the family *Potyviridae* (Gibbs and Ohshima 2010), is one of the most destructive plant pathogens of solanaceous crops. It causes substantial economic losses in global potato and tobacco production (Karasev and Gray 2013). PVY exists as a complex of strains and has been traditionally classified into biological strain groups, namely PVY^N^, PVY^O^, PVY^C^, PVY^Z^, and PVY^E^. The PVY^N^, PVY^C^, and PVY^O^ strain groups have been defined as non-recombinants (Singh et al. 2008), among which PVY^O^ and PVY^N^ are thought to serve as parents for the majority of recombinants (Ogawa et al. 2008, 2012; Hu et al. 2009; Gibbs et al. 2017). PVY^O^ and PVY^C^ induce hypersensitivity resistance in potato cultivars that harbour the *Nc*_tbr_ and *Ny*_tbr_ genes, respectively, whereas PVY^N^ has overcome these genes and does not induce hypersensitivity resistance in potato cultivars carrying them. However, PVY^N^ induces vein necrosis of tobacco, whereas PVY^O^ and PVY^C^ only induce vein mosaic symptoms (Karasev and Gray 2013). Additional groups identified by molecular classification include nine common and seven rare recombinants (Chikh-Ali et al. 2010; Green et al. 2017).

Phylogenetically, all PVY isolates can be clustered into five distinct phylogroups (N, C, O, R1, and R2) (Gibbs et al. 2017), which mostly correspond with the groupings based on plant responses. Isolates from the R1 and R2 phylogroups are all recombinants between isolates from the O and N phylogroups, and many of them cause tuber necrotic ringspot. Possibly owing to a fitness advantage over nonrecombinants, the recombinant strains now dominate in various regions of the world (Green et al. 2017; Green, Brown, and Karasev 2018; Bai et al. 2019).

PVY has a single-stranded, positive-sense RNA genome of 9.7 kb, which encodes a single polyprotein that is cleaved into ten mature proteins (King et al. 2011). In addition, a translational frameshift protein is encoded by the P3 cistron, which yields a P3N-PIPO fusion product (Chung et al. 2008). Among the 11 mature proteins, the genomic segments encoding P1 (the first protein), HC-Pro (helper-component proteinase), VPg (viral protein genome-linked), and CP (coat protein) are hotspots for recombination (Hu et al. 2009; Visser, Bellstedt, and Pirie 2012).

Based on a well-resolved phylogeny of 44 PVY complete genomic sequences (Visser, Bellstedt, and Pirie 2012), the most recent common ancestor (MRCA) was first dated to the 16th century CE (Common Era), which is when potato tubers were first taken from South America to Europe. An analysis of a dataset consisting of 73 non-recombinant complete ORF sequences (9,201 nt) yielded an estimate of 4553 BCE (Before the Common Era) to 602 CE for the MRCA (Gibbs et al. 2017). With the addition of 32 sequences from Peru, a more recent study, also of the complete ORFs, inferred that the MRCA of the current PVY population occurred 624 BCE–832 CE (Fuentes et al. 2019). However, an analysis of 176 VPg sequences (564 nt) placed the divergence between the N and O phylogroups at 1750 CE–1948 CE (Mao et al. 2019). Thus, there has been considerable variation among the date estimates reported for the MRCA of PVY.

There have been several reports of the likely migration pathways of potatoes (Francis 2005; Spooner et al. 2005; Mann 2011), but few reports on their viruses. To date, only the dispersal patterns of potato virus S (PVS), a carlavirus, have been investigated using Bayesian analyses of genetic data (Duan et al. 2018), and the global spread of PVY has been inferred cladistically from its phylogeny (Gibbs et al. 2017). Resolving the migration pathways of major pathogens of potato lays the foundation for understanding the patterns of causes of their geographic spread, with important implications for the protection of these crops.

In this study, we collected and analysed all of the sequence data from PVY isolates on GenBank. Using Bayesian phylodynamic methods, we analysed 253 non-recombinant sequences of partial P3, complete CI, and nearly complete NIb coding regions to produce a detailed picture of the migration pathways in PVY.

## 2. Materials and methods

### 2.1 Viral isolates and sequence dataset

Complete genomic sequences of 448 PVY isolates with known sampling dates and geographic locations were obtained from GenBank (12 June 2019, Supplementary Table S1). These isolates had been collected between 1938 and 2016 from 28 countries. To reduce computational complexity and increase post-analysis interpretability, we grouped the isolates into nine distinct geographic categories: Asia Mainland (AsM, *n *=* *39), Asian Island (AsI, *n *=* *18), Europe Island (EuI, *n *=* *21), Europe Mainland (EuM, *n *=* *58), Middle East (MdE, *n *=* *18), North America (NAm, *n *=* *232), Oceania (OcE, *n *=* *5), South Africa (SAF, *n *=* *49), and South America (SAm, *n *=* *8). We excluded one isolate from Peru with a long deletion in P1 (Czo25, accession number MH795871) and seven isolates from the countries that are represented by only a single viral isolate each. The dataset was unbalanced because 205 of the 440 PVY isolates were from the USA. To reduce this sampling bias, we randomly subsampled 30 of the 205 sequences from the USA, which provides a sufficient sample size for estimating the population genetic parameters. We assembled a total dataset consisting of 265 complete genomic sequences after down-sampling. The nucleotide sequences of the polyproteins were aligned using the codon-based MAFFT algorithm (Katoh and Standley 2013) implemented in PhyloSuite 1.16 (Zhang et al. 2020).

### 2.2 Recombination analysis

The full-length sequence alignment was screened for signals of recombination using the RDP 4.95 suite, which incorporates the algorithms RDP, GENECONV, BOOTSCAN, MAXCHI, CHIMAERA, SISCAN, and 3SEQ (Martin et al. 2015). For each putative recombination breakpoint, the sequences were analysed using the default settings. To minimize false identification, we only recognized recombination events that were detected by at least four of the seven methods, with an associated *P*-value of 10^−6^. Finally, we checked for recombination in the aligned sequences using the recombination analysis tool (RAT; Etherington, Dicks, and Roberts 2005). This analysis compared the percentage of nucleotide similarities using a sliding window of 30 nt, allowing detection of recombination breakpoints among sequences.

For all of our subsequent analyses, we selected three recombination-free protein-coding regions: partial P3 (P3*, nt 2,050–2,826, corresponding to the positions in the original genome), complete CI (nt 3,007–4,908), and nearly complete NIb (NIb*, nt 6,358–7,668). Each of these three datasets comprised sequences from 253 non-recombinant PVY isolates after we removed the recombinant sequences and any isolate that did not have sequence data for all three protein-coding regions (Supplementary Table S2). To test for the presence of reticulate evolution in the PVY genome, we performed a phylogenetic network analysis of a concatenated P3*, CI, and NIb* alignment using the Neighbor-Net method in SplitsTree 4.13.1 (Huson 1998).

### 2.3 Tests for temporal signal

To assess the temporal structure in the sequence data, we regressed phylogenetic root-to-tip distances against date of sampling using TempEst 1.5 (Rambaut et al. 2016). For this analysis, we inferred the tree topology and branch lengths using maximum likelihood under the best-fit substitution model, which was selected using the Bayesian information criterion by ModelFinder (Kalyaanamoorthy et al. 2017) implemented in PhyloSuite (Zhang et al. 2020). Each regression yielded a low *r*^2^ value, indicating the presence of rate heterogeneity among lineages (Supplementary Fig. S1). Our plots also showed that the O3 subgroup of PVY had an unusual temporal signal, which we took into account in some of our subsequent analyses.

Bayesian dating analyses of time-structured datasets can be misled when the samples have been drawn from a highly structured population. To reduce the potential impacts of genetic structure on molecular dating (Murray et al. 2016), we performed a Mantel test of the correlation between pairwise genetic distances and differences in sampling dates and calculated the *P*-value of this test with 1,000 permutations. The temporal and genetic structure was only confounded in the NIb* dataset (*P *<* *0.05, Supplementary Fig. S2A). We then performed a clustered date-permutation test, in which the temporal signal was evaluated by randomizing the sampling dates over clusters of tips and not over individual tips (Duchêne et al. 2015). The mean substitution rate estimated from the real sampling dates did not overlap with the 95 per cent credibility intervals of rate estimates from ten replicate datasets with cluster-permuted sampling dates (Supplementary Fig. S2B). Our results confirmed the presence of temporal structure in the sequence data, allowing us to proceed with our Bayesian molecular dating analyses.

### 2.4 Temporal dynamics of PVY

To infer the evolutionary rate and timescale of PVY, we analysed datasets containing 161 dated non-recombinant nucleotide sequences for the complete polyprotein (Fuentes et al. 2019) and 253 dated non-recombinant sequences of P3*, CI, and NIb*. From the 161 sequences of the complete polyprotein, we constructed additional datasets that were partially concatenated or partitioned in order to investigate whether there were any region-dependent effects on the estimates. We also analysed a 154 sequences dataset, from which we removed seven isolates of the O3 subgroup based on our investigations of temporal signal. We performed the analyses in a Bayesian framework implemented in the software package BEAST 1.10 (Suchard et al. 2018). The best-fit model of nucleotide substitution was chosen for each dataset as described above. We computed marginal likelihoods using path sampling and stepping-stone sampling (Baele et al. 2012) to compare the constant-size, exponential-growth, and Bayesian skyline coalescent tree priors, and to compare the strict molecular clock and uncorrelated lognormal relaxed clock (Drummond et al. 2006).

All analyses were run for 10^8^ steps across three independent Markov chain Monte Carlo (MCMC) simulations and states were sampled every 10^4^ steps. We used Tracer 1.71 (Rambaut et al. 2018) to check for sufficient sampling and convergence among the three sets of samples, after discarding the first 10 per cent of samples as burn-in. The maximum-clade-credibility trees were summarized using TreeAnnotator, part of the BEAST package.

### 2.5 Discrete phylogeographic analysis

To investigate the spatial dynamics of the virus, we performed phylogeographic analysis using an asymmetric continuous-time Markov chain with Bayesian stochastic search variable selection implemented in BEAST (Lemey et al. 2009). To ensure temporal and geographic representation, we analysed the 253 dated recombination-free sequences of P3, CI, and NIb. Nine geographical regions, as described above, were chosen and coded as discrete states. We considered pairwise diffusions be important when the Bayes factor (BF) was >3 and when the posterior probability of the corresponding node was >0.50. Diffusion pathways were summarized in SPREAD3 0.9.7 (Bielejec et al. 2016). We also calculated the number of expected location-state transitions (Markov jump counts) based on the reconstructed phylogeny (Minin and Suchard 2008).

## 3. Results

### 3.1 Recombination analyses

Significant signals of recombination were found in the 448 polyprotein sequences and the partial genomic sequences of 447 P3, 446 CI, and 440 NIb coding-regions in our analyses using RDP4 and RAT. Both showed that the two major recombination sites in PVY genomes found in earlier studies (Ogawa et al. 2008; Green et al. 2017, Green, Brown, and Karasev 2018), which approximately correspond to the boundaries of the HC-Pro and P3 protein-coding regions and in the N-terminus of the VPg protein-coding region, were also found in the present study (Supplementary Fig. S3). Furthermore, we found some novel unequivocal recombination sites in the P3, CI, and NIb coding-region sequences, but all are likely to be minor. The common recombination site found in the P3-coding region was in isolates from Japan, UK, Canada, USA, and South Africa with Colombian and Peruvian parents, and those were N intralineage recombinants. The most common recombination site found in the NIb-coding region was in isolates from China and Syria with Peruvian and US parents, and those were N and O interlineage recombinants. We removed these recombinants from our datasets, and subsequently analysed P3, CI, and NIb coding sequences from the same 253 isolates.

The 162 ORF sequences from the study by Fuentes et al. (2019) were re-checked for evidence of recombination. One clear recombination site was found in the helper-component proteinase protein gene of isolate Czo29 (accession number MH795870, Supplementary Table S3).

### 3.2 Evolutionary rates and timescales

**Figure 1.**
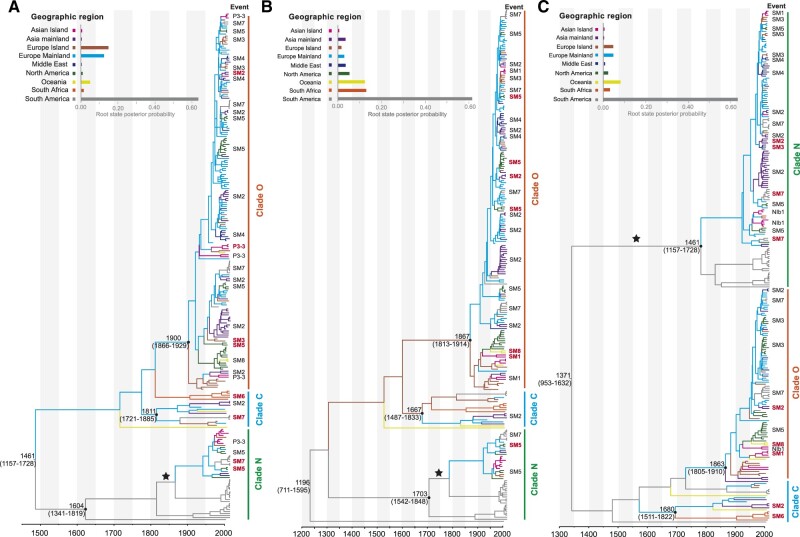
Time-scaled maximum-clade-credibility trees inferred from the protein-coding genes P3*(A), CI (B), and NIb* (C). The tree topologies have been chosen to maximize the product of node posterior probabilities. Black dots indicate the most recent common ancestors of the Clades N, O, and C, which have >0.95 posterior probability. Branch lengths are scaled according to time (year CE), as shown by the horizontal axis. Branch colours denote inferred location states. The root state posterior probabilities of the geographic regions are shown in each inset panel. Identified migration events are shown next to the tips, and bold red font indicates migration events supported by Bayes factors. SM# indicates shared migration events inferred from all three protein-coding genes. Introduction of potato virus Y into Europe Mainland from South America is marked with a star in each tree.

Bayesian skyline coalescent tree priors and uncorrelated lognormal relaxed clocks provided the best fit to all of the datasets (Supplementary Table S4). For the dataset comprising 253 sequences from each of the P3*, CI, and NIb* segments, our time-scaled maximum-clade-credibility tree showed that all PVY isolates could be separated into three major clades, corresponding to the well-established Clades N, O, and C (Fig. 1). The maximum-clade-credibility trees inferred from P3* and CI shared very similar topologies, but these were highly divergent from that inferred from NIb*, suggesting the presence of reticulate evolution in the PVY genome. The results of our phylogenetic network analysis also suggest that the three segments have conflicting phylogenetic signals (Supplementary Fig. S4). Our Bayesian analysis places the root of the trees in South America with high posterior probability (>0.50, Fig. 1). The MRCAs were dated to 1461 CE (95% credibility interval: 1157 CE–1728 CE) for P3*, 1196 CE (95% credibility interval: 711 CE–1595 CE) for CI, and 1317 CE (95% credibility interval: 953 CE–1632 CE) for NIb*.

The mean substitution rates of P3*, CI, and NIb* estimated for the 253 dated (with discrete location states) recombination-free sequences were 2.06 × 10^−4^ subs/site/year (95% credibility interval: 1.41 × 10^−4^ to 2.71 × 10^−4^), 1.49 × 10^−4^ subs/site/year (95% credibility interval: 1.00 × 10^−4^ to 2.03 × 10^−4^), and 1.57 × 10^−4^ subs/site/year (95% credibility interval: 1.14 × 10^−4^ to 2.02 × 10^−4^), respectively (Supplementary Table S5). The 95 per cent credibility intervals overlapped for the estimates of MRCA dates and substitution rates from the three genome segments.

We also analysed several PVY datasets to infer the date of the MRCA of global PVY (Supplementary Fig. S5 and Table S5). The estimated date of the MRCA from the 161-sequence ORF dataset was 115 CE (95% credibility interval: 675 BCE–844 CE). In addition, the mean date of the MRCA of the 154 ORFs, after removal of seven isolates of the O3 subgroup, was 70 CE (95% credibility interval: 808 BCE–822 CE). The estimated time of the MRCA from the P3* + CI + NIb* (partitioned) genomic sequences of 161 isolates was 289 CE (95% credibility interval: 544 BCE–1033 CE).

### 3.3 Global migration pattern of PVY

We firstly used the 161 dated recombination-free polyprotein nucleotide sequences for our phylogeographic analysis. Although this dataset passed the cluster-based date-randomization test, we encountered serious problems with MCMC convergence for the parameters relating to spatial diffusion (effective sample sizes below 200). This result indicated that the dataset comprising 161 polyprotein nucleotide sequences was unsuitable for phylogeographic analysis. Therefore, we used our dataset containing 253 non-recombinant P3, CI, and NIb coding sequences to estimate the migration pathways. We anticipated that these larger datasets would reveal the migration history more effectively.

**Figure 2.**
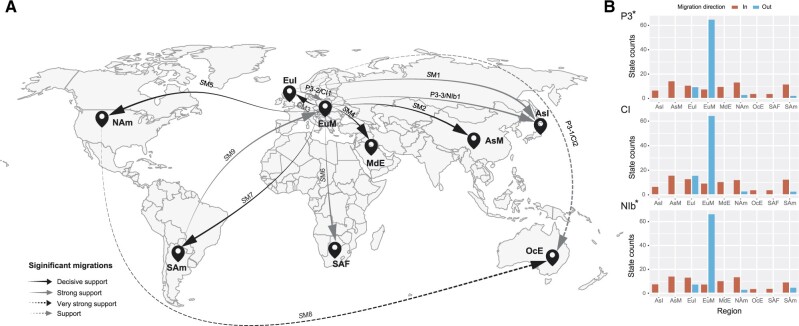
Spatial diffusion of potato virus Y. (A) Supported spatial diffusion pathways and (B) histogram of the total number of location-state transitions inferred from P3*, CI, and NIb*. Migration events presented in Table 1 are shown above the arrows. Solid black arrows indicate decisively supported diffusions (BF > 1000); dashed black arrows, very strongly supported diffusions (150 < BF < 1000); solid grey arrows, strongly supported diffusions (20 < BF < 150); and dashed grey arrows, supported diffusions (3 < BF < 20). AsI, Asian Island; AsM, Asia Mainland; EuI, Europe Island; EuM, Europe Mainland; MdE, Middle East; NAm, North America, OcE, Oceania; SAF, South Africa; and SAm, South America.

Nine migration pathways in the spatial diffusion of PVY were supported by our Bayesian phylogeographic analysis of P3*, CI, and NIb* coding regions. Six originated from Europe Mainland to Asia Mainland, Europe Island, Middle East, North America, South Africa, and South America, and other three from Europe Island to Asian Island, North America to Oceania, and South America to Europe Mainland (Fig. 2A;  Supplementary Table S6). Additional diffusions from Europe Island to Europe Mainland and Oceania were supported by both the P3* and CI datasets, and from Europe Mainland to Asian Island by both the P3* and NIb datasets (Supplementary Table S6).

The inferred spatial dynamics of PVY suggest that Europe Mainland has acted as an important source for the epidemics in other regions. This was also supported by the state-change counts, with migration from Europe Mainland being much greater than from any other geographic region included in our analysis (Fig. 2B). In addition, the mean migration rate and Markov jump counts (Supplementary Fig. S6) supported the occurrence of multiple migrations between Europe Mainland and other geographic regions.

**Figure 3.**
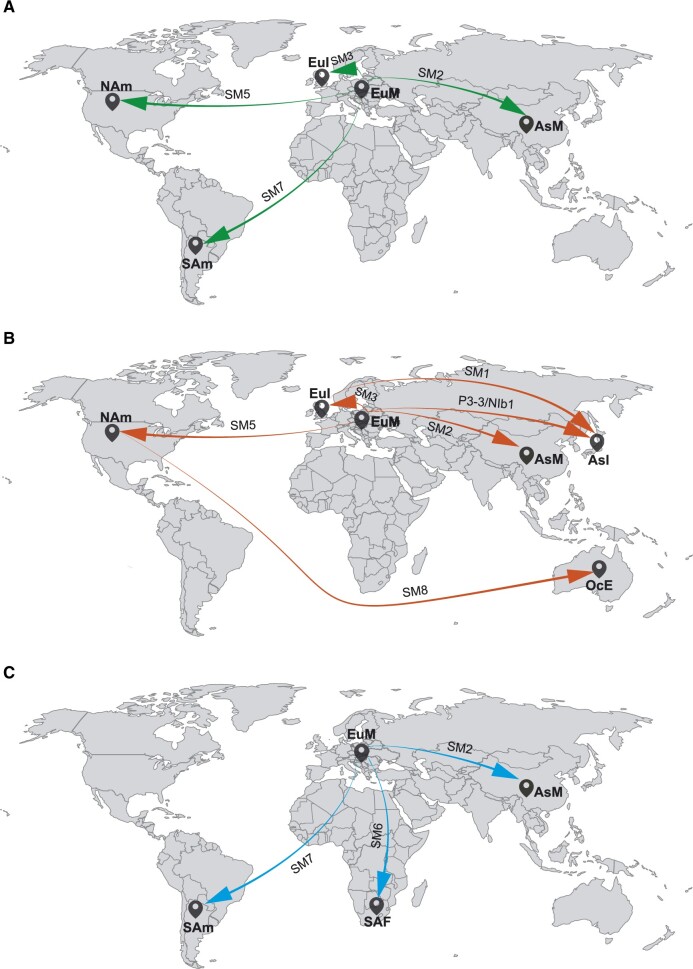
Global spatial diffusion of Clade N (A), Clade O (B), and Clade C (C) of potato virus Y. Migration pathways from different clades are indicated by a unique colour. For each clade, supported migrations presented in Table 1 are shown above the arrows. AsI, Asian Island; AsM, Asia Mainland; EuI, Europe Island; EuM, Europe Mainland; NAm, North America, OcE, Oceania; SAF, South Africa; and SAm, South America.

For Clade N, four pathways were apparent, generally indicating migration from Europe Mainland to Asia Mainland, Europe Island, North America, and South America (Fig. 3A). Migration patterns for Clade O are similar to those of Clade N, with five migration links originating from other regions (Asian Island, Asia Mainland, Europe Island, North America, and South America) and one from North America to Oceania (Fig. 3B). For Clade C, three migration pathways were recognized from Europe Mainland to Asia Mainland, South America, and South Africa (Fig. 3C). These migrations were supported by data from at least one of the P3*, CI, and NIb* coding regions, but the migration link from Europe Mainland to Asia Mainland for the O clade was supported by all three datasets (Table 1). In total, the results of our Bayesian phylogeographic analysis suggest that Europe Mainland was an important hub for the global spread of PVY.

**Table 1.** Migration events of potato virus Y strains inferred from P3*, CI, and NIb* coding regions.

Event	Migration		N strain			O strain			C strain		
	From	To	P3	CI	NIb	P3	CI	NIb	P3	CI	NIb
SM1	EuI	AsI	−	−	−	−	+***	+**	−	−	−
SM2	EuM	AsM	−	−	+****	+****	+ ****	+****	−	−	+ ****
SM3	EuM	EuI	−	−	+ ****	+****	−	−	−	−	−
SM4	EuM	MdE	−	−	−	−	−	−	−	−	−
SM5	EuM	NAm	+****	+****	−	+ ****	+ ****	−		−	−
SM6	EuM	SAF	−	−	−	−	−	−	+**	−	+**
SM7	EuM	SAm	+****	−	+ ****	−	−	−	+ ****	−	−
SM8	NAm	OcE	−	−	−	−	+ *	+ **	−	−	−
SM9	SAm	EuM	−	−	−	−	−	−	−	−	−
P3-1/CI2	EuI	OcE	−	−	NA	−	−	NA	−	−	NA
P3-2/CI1	EuI	EuM	−	−	NA	−	−	NA	−	−	NA
P3-3/NIb1	EuM	AsI	−	NA	−	+**	NA	−	−	NA	−

SM#, shared migration identified simultaneously by the datasets of P3*, CI and NIb*; P3-#/CI# indicates migration identified by the datasets of P3*and CI; P3-#/NIb# indicates migration identified by the datasets of P3*and Nib; +, supported migration event; −, unsupported migration event; NA, no data available; AsM, Asia Mainland; AsI, Asian Island; EuI, Europe Island; EuM, Europe Mainland; MdE, Middle East; NAm, North America; OcE, Oceania; SAF, South Africa; SAm, South America.

Statistically supported migrations are marked with asterisks: *3 < BF < 20; **20 < BF < 150; ***150 < BF < 1000; ****BF > 1000.

### 3.4 Demographic history of PVY

**Figure 4.**
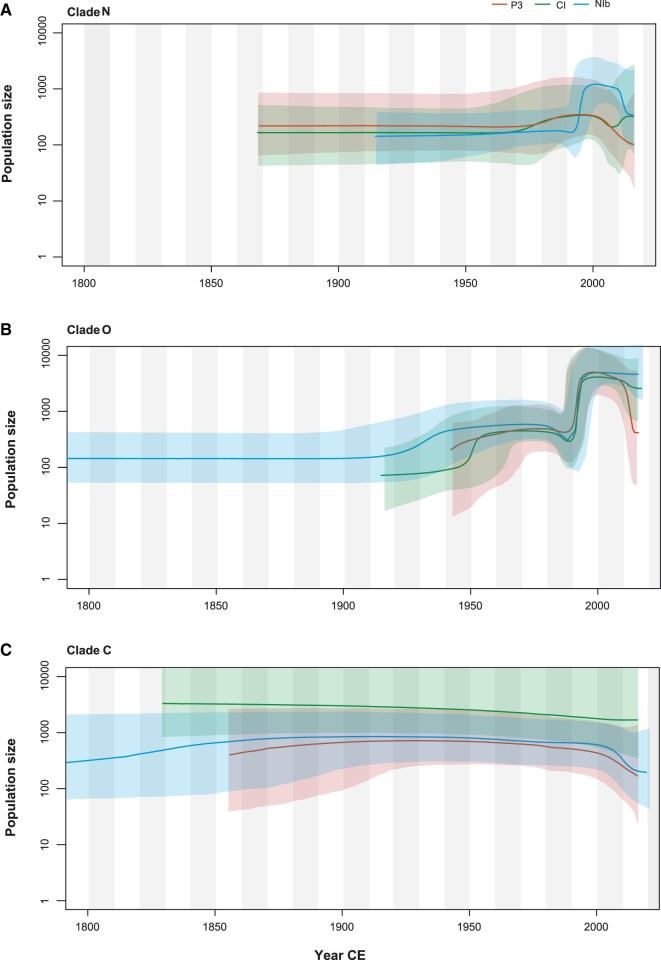
Bayesian skyline plots of Clade N (A), Clade O (B), and Clade C (C) of potato virus Y inferred from P3*(in orange), CI (in green), and NIb*(in blue), showing population size (*y*-axis) through time (*x*-axis). Solid lines represent the median estimates of population size and the shaded areas indicate the corresponding 95 per cent credibility intervals.

Bayesian skyline plots depicted similar demographic histories for the PVY populations of Clade N, O, and C based on the P3*, CI, and NIb* data (Fig. 4). The population of Clade N underwent a short, slight expansion prior to a period of stability, followed by a recent population decline. PVY from Clade O maintained a steady population size with a slight increase until about 1990, then experienced a sudden population expansion. In contrast with the patterns in Clades N and O, Clade C has maintained a constant population size through time.

## 4. Discussion

In this study, we assessed the evolutionary and global transmission dynamics of PVY using Bayesian phylodynamic inference. Previous work found that most of the viruses (e.g. wild potato mosaic virus, Peru tomato virus, Bidens mosaic virus, sunflower ring blotch virus, and potato virus V) that are most closely related to PVY (i.e. the PVY lineage) were isolated from plants that are native to South America (Gibbs and Ohshima 2010; Fribourg et al. 2019). Our phylogenetic analysis placed the root of the lineage of PVY in South America with strong support, providing further evidence of the geographic origins of PVY.

We found strong support for a migration pathway from South America to Europe Mainland in the 19th century. However, our Bayesian phylogeographic analysis revealed Europe as an important source of PVY epidemics in the world. This can be explained by a scenario in which PVY was firstly introduced into Europe from South America, then established strong links with multiple geographic regions in the subsequent global spread. This pattern is likely to be associated with the global trade of potatoes. For example, some European countries, such as the Netherlands and Poland, produce substantial exports of seed potatoes that are used for planting. Our Bayesian coalescent analyses also showed that the one introduction of PVY to Australia might have been from North America. Many potato seeds as breeding sources are imported to Australia from countries that include the USA and Canada (Slater 2016).

Strain PVY^C^, which causes less severe disease symptom in potato cultivars with stipple streak or systemic mosaic symptoms, was considered to be less prevalent. In the past decades, however, N × O recombinant isolates have become prevalent throughout the cultivated potato regions of Europe, North America, and other parts of the world (Quenouille, Vassilakos, and Moury 2013; Lacomme et al. 2017). This is consistent with the results of our demographic analyses, which showed that the population size of Clade C has remained relatively constant. In contrast, Clades N and O experienced similar population expansions in the past few decades prior to a more recent decline. This is in accordance with a recent demographic analysis by Mao et al. (2019), which suggested that the population of Clade N was associated with the global history of potato production.

Our estimated dates of the MRCA of the 161 and 154 dated recombination-free nucleotide sequences for the polyprotein are similar to the recent date estimate for the MRCA of 137 CE (95% credibility interval: 644 BCE–824 CE) by Fuentes et al. (2019). When the dates of the MRCAs were estimated using 161 and 253 sequences for P3, CI, and NIb protein-coding regions, the number of sequences appeared to have some influence. We found that partial genomic and polyprotein regions might lead to different MRCA estimates (Supplementary Fig. S5 and Table S5). The 253 isolates that we used for MRCA estimates have recombination-free sequences, but some might be recombinants when viewed at the whole-genome scale. Specifically, the isolate sequences might have recombination sites between the P3, CI, and NIb protein-coding regions used in this study. The presence of such ‘hidden recombinants’ might affect the date estimates for the MRCA. On the other hand, the 161 PVY polyprotein sequences used in this study are not yet representative and provide only a relatively small number of sequences for estimating the date of the MRCA. The polyprotein sequences include many isolates from the USA and from a limited set of countries, and the 161 sequences might also contain ‘recombinants’. Further investigation is needed to confirm our estimates, using larger collections of the parental isolates sampled on a worldwide scale.

We found that our date estimates for the MRCA differed when the sampling locations of the isolates were taken into account in the analysis (Supplementary Table S5). To date, one of the difficulties of estimating the dates of MRCA is that plant RNA viruses contain many recombinants, so reliable estimation of dates requires larger samples of sequences. Our study has revealed discrepancies in date estimates from PVY partial genomic and polyprotein region sequences, a problem that will potentially be resolved in future studies using larger datasets. 

Our study has provided new insights into the evolutionary history of PVY. In particular, we found multiple migration pathways of PVY from Europe to other regions, with the European mainland having been an important hub for the global spread of this pathogen. A similar observation has been made for PVS (Duan et al. 2018). This suggests that, as a hub for potato propagation from South America to the rest of the world, Europe has also been responsible for the spread of many viruses of this crop plant. These dispersals are likely to be related to human activities, including seed potato exchange and other agricultural practices. Our findings provide epidemiological evidence relating to dispersal patterns of PVY, which could be implemented in the design for PVY control strategies. However, challenges remain to inferring the evolutionary scenarios of PVY involving recombinants. Further investigations using methods that can account for recombinants, including those based on phylogenetic networks, will provide a more comprehensive view of the evolutionary history of PVY.

## Data availability

Data are available through Dryad. 

## Supplementary data

Supplementary data are available at *Virus Evolution* online.

## Supplementary Material

veaa056_Supplementary_Data
