# Supplementary material for: The evolutionary history and global spatio-temporal dynamics of potato virus Y
Source: Virus Evol. 2020 Nov 21;6(2):veaa056. doi: 10.1093/ve/veaa056 (PMC7724251; doi:10.1093/ve/veaa056)
Supplement: veaa056_Supplementary_Data [file veaa056_supplementary_data.zip › suppl_data/Table_S3_R1.docx]

**Table S3** Recombination site in potato virus Y Czo29 isolate genome.

| Isolate | Country | Coding region | Recombination site | Parental sequence | | Recombination detecting program (*p*-value) | | | | | |
| --- | --- | --- | --- | --- | --- | --- | --- | --- | --- | --- | --- |
|  |  |  | Position | Major | Minor | RDP | GENECONV | BOOTSCAN | MAXCHI | CHIMAERA | SISCAN |
| Czo29  (MH795870) | Peru | HC-Pro | 2229 | SCRI-O  (AJ585196) | Jin125  (MH795861) | 1.38×10^-10^ | 1.79×10^-12^ | 6.05×10^-12^ | 1.12×10^-5^ | 7.43×10^-5^ | 2.33×10^-15^ |

The estimated nucleotide position of the recombination site is shown relative to the 5'-end of the genome using the numbering of the sequence of the Ditta isolate (Schubert et al., 2007).
