# Supplementary material for: The evolutionary history and global spatio-temporal dynamics of potato virus Y
Source: Virus Evol. 2020 Nov 21;6(2):veaa056. doi: 10.1093/ve/veaa056 (PMC7724251; doi:10.1093/ve/veaa056)
Supplement: veaa056_Supplementary_Data [file veaa056_supplementary_data.zip › suppl_data/Table_S4_RF.docx]

**Table S4** Marginal likelihoods of different combinations of clock model and tree prior

| **Protein-coding region** | **Molecular clock model** | **Coalescent tree prior** | **Log marginal likelihood** |
| --- | --- | --- | --- |
| P3 | Strict clock | Bayesian skyline | -9509.112 |
|  | Strict clock | Constant size | -9531.599 |
|  | Strict clock | Exponential growth | -9525.295 |
|  | **Uncorrelated lognormal relaxed clock** | **Bayesian skyline** | **-9483.910** |
|  | Uncorrelated lognormal relaxed clock | Constant size | -9485.400 |
|  | Uncorrelated lognormal relaxed clock | Exponential growth | -9512.938 |
| CI | Strict clock | Bayesian skyline | -21271.786 |
|  | Strict clock | Constant size | -21300.066 |
|  | Strict clock | Exponential growth | -21305.914 |
|  | **Uncorrelated lognormal relaxed clock** | **Bayesian skyline** | **-21154.513** |
|  | Uncorrelated lognormal relaxed clock | Constant size | -21216.755 |
|  | Uncorrelated lognormal relaxed clock | Exponential growth | -21180.633 |
| NIb | Strict clock | Bayesian skyline | -15410.178 |
|  | Strict clock | Constant size | -15430.013 |
|  | Strict clock | Exponential growth | -15434.042 |
|  | **Uncorrelated lognormal relaxed clock** | **Bayesian skyline** | **-15366.131** |
|  | Uncorrelated lognormal relaxed clock | Constant size | -15371.246 |
|  | Uncorrelated lognormal relaxed clock | Exponential growth | -15376.490 |

The best-fitting tree prior and molecular clock model are indicated in bold font.
