# Supplementary material for: The evolutionary history and global spatio-temporal dynamics of potato virus Y
Source: Virus Evol. 2020 Nov 21;6(2):veaa056. doi: 10.1093/ve/veaa056 (PMC7724251; doi:10.1093/ve/veaa056)
Supplement: veaa056_Supplementary_Data [file veaa056_supplementary_data.zip › suppl_data/Table_S5_RF.docx]

Table S5. Timescale analysis of potato virus Y using different protein-coding regions^a^

| **Number of sequences** | **Region analysed^b^** | **Sequence**  **length (nt)** | **MRCA^c^ (Year)** | **Substitution rate (substitutions/site/year)** | **Reference** |
| --- | --- | --- | --- | --- | --- |
| n=162 ^d^ | Polyprotein (From P1 to CP) | 8913 | 137CE (644BC–824CE) | 9.16×10^-5^ (6.90×10^-5^–1.15×10^-4^) | Fuentes et al. (2019) |
| n=161 | Polyprotein (From P1 to CP) | 8913 | 115CE (675BC–844CE) | 8.94×10^-5^ (6.63×10^-5^–1.14×10^-4^) | This study |
| n=161 ^e^ | From HC-Pro to CP (concatenated) | 8358 | 208CE (595BC–892CE) | 9.00×10^-5^ (6.47×10^-5^–1.15×10^-4^) | This study |
|  | From P1 to NIb (concatenated) | 8112 | 205CE (570BC–870CE) | 9.39×10^-5^ (7.19×10^-5^–1.18×10^-4^) | This study |
|  | From HC-Pro to NIb (concatenated) | 7557 | 246CE (541BC–955CE) | 9.46×10^-5^ (7.12×10^-5^–1.20×10^-4^) | This study |
|  | From CI to CP (concatenated) | 6963 | 316CE (485BC–1000CE) | 9.34×10^-5^ (6.72×10^-5^–1.23×10^-4^) | This study |
|  | From CI to NIb (concatenated) | 6162 | 404CE (394BC–1079CE) | 1.01×10^-4^ (7.27×10^-5^–1.31×10^-4^) | This study |
|  | Partial P3 + CI+partial NIb (partitioned) | 3996 | 289CE (544BC–1033CE) | 8.99×10^-5^ (6.45×10^-5^–1.19×10^-4^) | This study |
|  | Partial P3 + CI+partial NIb (concatenated) | 3996 | 279CE (491BC–1042CE) | 8.95×10^-5^ (6.46×10^-5^–1.18×10^-4^) | This study |
|  | Partial P3 + CI+partial NIb (concatenated) (silent sites) ^f^ | 2676 | 287CE (566BC–1046CE) | 1.07×10^-4^ (7.59×10^-5^–1.38×10^-4^) | This study |
|  | Partial P3 + CI (partitioned) | 2679 | 333CE (558BC–1092CE) | 9.98×10^-5^ (7.03×10^-5^–1.30×10^-4^) | This study |
|  | Partial CI + partial NIb (partitioned) | 3213 | 463CE (244BC–1083CE) | 1.02×10^-4^ (7.31×10^-5^–1.33×10^-4^) | This study |
|  | Partial P3 + partial NIb (partitioned) | 2088 | 629CE (136BC–1299CE) | 1.07×10^-4^ (7.13×10^-5^–1.46×10^-4^) | This study |
|  | Partial P3 | 777 | 934CE (139CE–1579CE) | 1.34×10^-4^ (8.09×10^-5^–1.96×10^-4^) | This study |
|  | CI | 1902 | 424CE (378BC–1130CE) | 1.12×10^-4^ (7.67×10^-5^–1.51×10^-4^) | This study |
|  | Partial NIb | 1311 | 725CE (123CE–1250CE) | 1.13×10^-4^ (7.39×10^-5^–1.52×10^-4^) | This study |
| n=253 | Partial P3 | 777 | 942CE (313CE–1489CE) | 1.62×10^-4^ (1.14×10^-4^–2.13×10^-4^) | This study |
|  | CI | 1902 | 103CE (1056BC–1039CE) | 1.10×10^-4^ (8.44×10^-5^–1.38×10^-4^) | This study |
|  | Partial NIb | 1311 | 564CE (329BC–1309CE) | 1.16×10^-4^ (8.38×10^-5^–1.50×10^-4^) | This study |
|  | Partial P3 (with discrete location states ^h^) | 777 | 1461CE (1157CE–1728CE) | 2.06×10^-4^ (1.41×10^-4^–2.71×10^-4^) | This study |
|  | CI (with discrete location states) | 1902 | 1196CE (711CE–1595CE) | 1.49×10^-4^ (1.00×10^-4^–2.03×10^-4^) | This study |
|  | Partial NIb (with discrete location states) | 1311 | 1317CE (953CE–1632CE) | 1.57×10^-4^ (1.14×10^-4^–2.02×10^-4^) | This study |
| n=28 | Polyprotein (from P1 to CP) | 9723 | Unknown (1391CE-1849CE  /1574CE-1887CE/1040CE-1485CE) ^g^ | Unknown | Visser et al. (2012) |
| n=176 | VPg | 564 | 1755CE (1517CE–1929CE) | 4.41×10^-4^ (2.41×10^-4^–6.41×10^-4^) | This study |
|  | VPg (with discrete location states) | 564 | 1861CE (1750CE–1948CE) | 5.60×10^-4^ (3.35×10^-4^–8.17×10^-4^) | Mao et al. (2019) |

^a^ GTR+I+Γ_4_ substitution model, uncorrelated lognormal relaxed clock, and Bayesian skyline coalescent model were used.

^b^ The regions were analysed using total sites.

^c^ All data sets passed date-randomization tests except the data set from Visser et al. (2012). MRCA, most recent common ancestor.

^d^ One clear recombinant included.

^e^ One clear recombinant was removed from 162 isolates before analysis.

^f^ Silent sites were analysed.

^g^ Different clock models were used.

^h^ Discrete location states in parentheses indicate discrete traits incorporated in Bayesian phylogeographic analysis to infer the ancestral locations of the virus.
