# Supplementary material for: The evolutionary history and global spatio-temporal dynamics of potato virus Y
Source: Virus Evol. 2020 Nov 21;6(2):veaa056. doi: 10.1093/ve/veaa056 (PMC7724251; doi:10.1093/ve/veaa056)
Supplement: veaa056_Supplementary_Data [file veaa056_supplementary_data.zip › suppl_data/Table_S6_RF.docx]

**Table S6** Statistically supported migration rates of potato virus Y

| **Protein-coding region** | **Migration event** | **From** | **To** | **Indicator ^a^** | **Bayes factor ^b^** |
| --- | --- | --- | --- | --- | --- |
| P3 | SM1 | EuI | AsI | 15.50 | 0.68 |
|  | SM2 | EuM | AsM | >1000 | 1.00 |
|  | SM3 | EuM | EuI | >1000 | 1.00 |
|  | SM4 | EuM | MdE | >1000 | 1.00 |
|  | SM5 | EuM | NAm | >1000 | 1.00 |
|  | SM6 | EuM | SAF | 70.57 | 0.91 |
|  | SM7 | EuM | SAm | >1000 | 1.00 |
|  | SM8 | NAm | OcE | 103.26 | 0.93 |
|  | SM9 | SAm | EuM | 21.24 | 0.74 |
|  | P3-1 | EuI | OcE | 11.69 | 0.62 |
|  | P3-2 | EuI | EuM | 27.33 | 0.79 |
|  | P3-3 | EuM | AsI | 35.39 | 0.83 |
| CI | SM1 | EuI | AsI | 179.83 | 0.96 |
|  | SM2 | EuM | AsM | >1000 | 1.00 |
|  | SM3 | EuM | EuI | >1000 | 1.00 |
|  | SM4 | EuM | MdE | >1000 | 1.00 |
|  | SM5 | EuM | NAm | >1000 | 1.00 |
|  | SM6 | EuM | SAF | 43.16 | 0.86 |
|  | SM7 | EuM | SAm | >1000 | 1.00 |
|  | SM8 | NAm | OcE | 11.04 | 0.60 |
|  | SM9 | SAm | EuM | 9.48 | 0.57 |
|  | CI1 | EuI | EuM | 42.22 | 0.85 |
|  | CI2 | EuI | OcE | 42.12 | 0.85 |
| NIb | SM1 | EuI | AsI | 69.92 | 0.91 |
|  | SM2 | EuM | AsM | >1000 | 1.00 |
|  | SM3 | EuM | EuI | >1000 | 1.00 |
|  | SM4 | EuM | MdE | >1000 | 1.00 |
|  | SM5 | EuM | NAm | >1000 | 1.00 |
|  | SM6 | EuM | SAF | 45.11 | 0.86 |
|  | SM7 | EuM | SAm | >1000 | 1.00 |
|  | SM8 | NAm | OcE | 56.61 | 0.89 |
|  | SM9 | SAm | EuM | 44.58 | 0.86 |
|  | NIb1 | EuM | AsI | 26.14 | 0.78 |

SM indicates shared migration event identified simultaneously by the datasets of P3^*^, CI and NIb^*^.

^a^ Posterior probability of observing a non-zero migration rate in the sampled trees.

^b^ Only statistically supported migrations with indicator values >0.50 and Bayes factor >3 are shown.
