# Supplementary figures and images for: The evolutionary history and global spatio-temporal dynamics of potato virus Y
Source: Virus Evol. 2020 Nov 21;6(2):veaa056. doi: 10.1093/ve/veaa056 (PMC7724251; doi:10.1093/ve/veaa056)

**Fig. S1** polyprotein

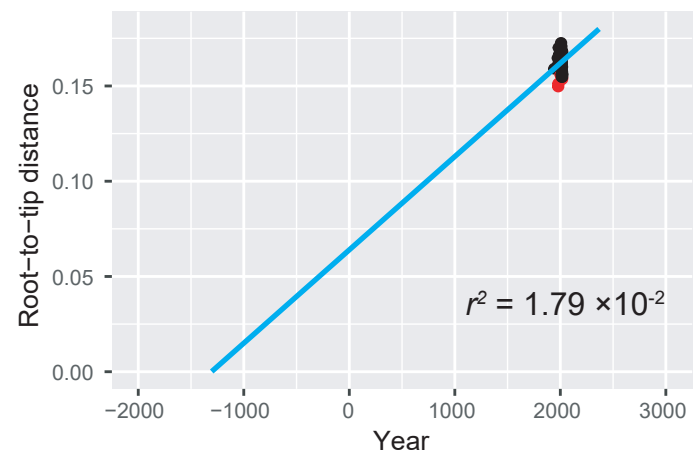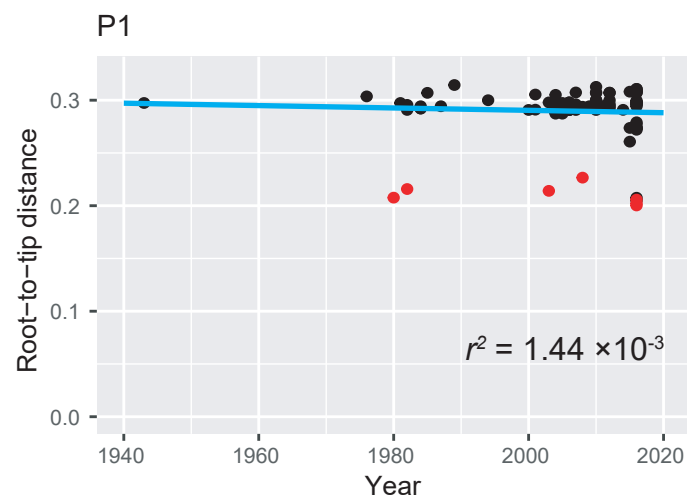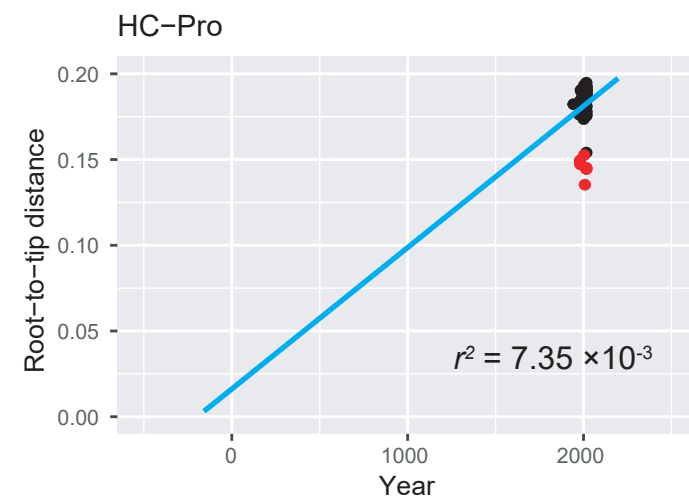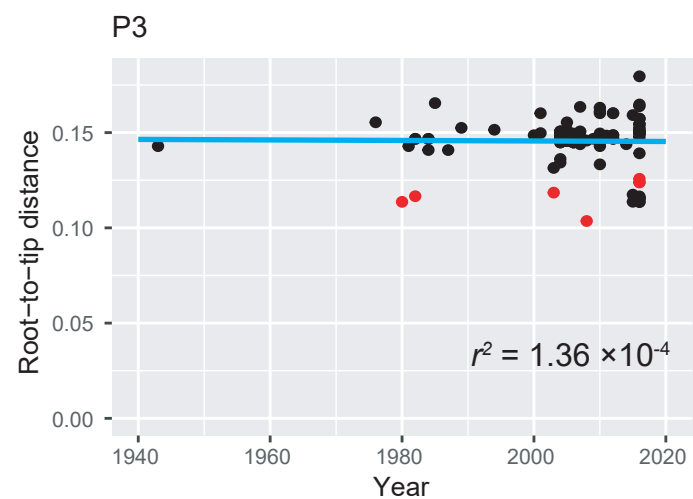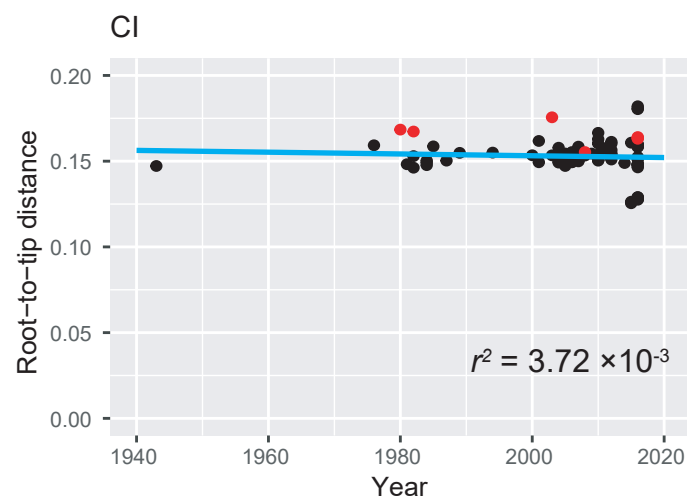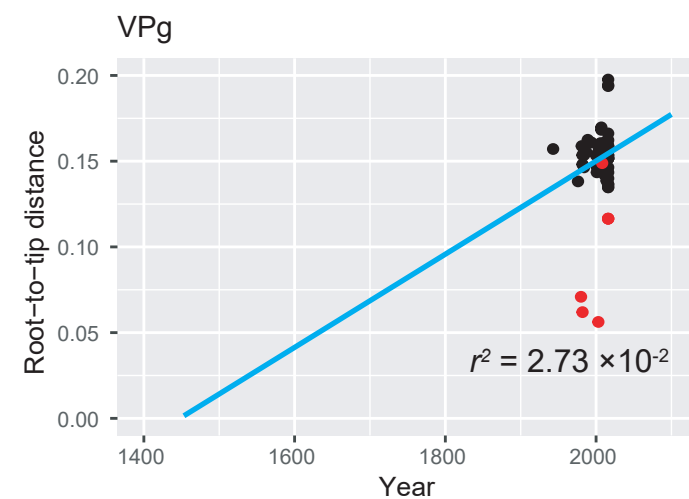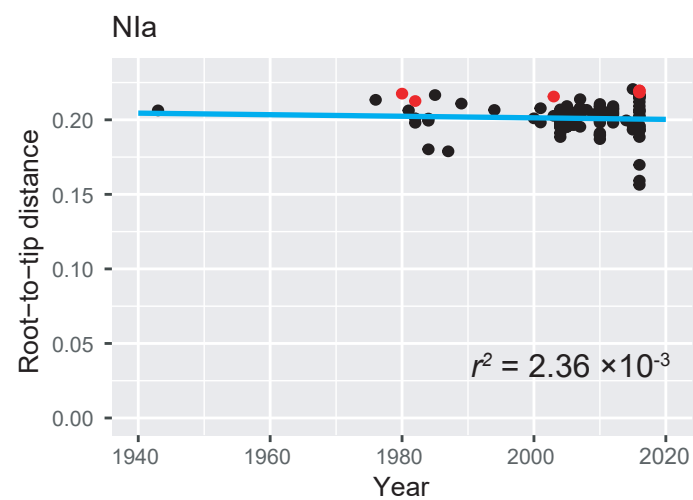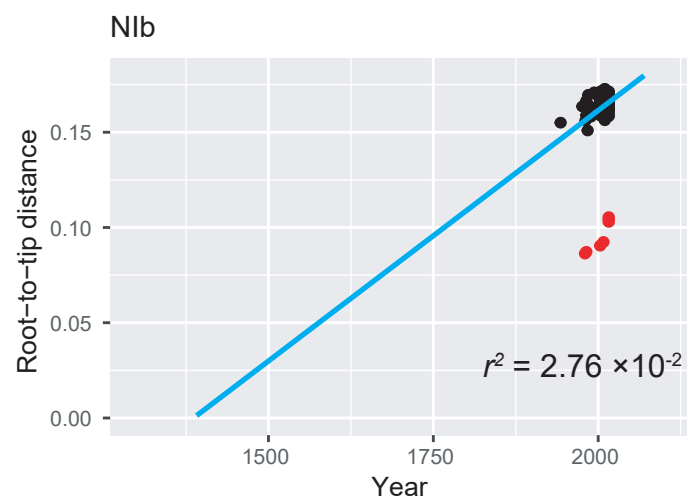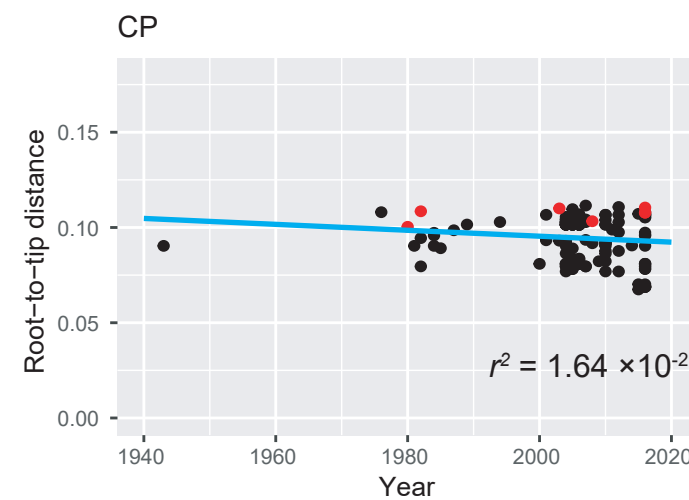

Supplement: veaa056_Supplementary_Data [file veaa056_supplementary_data.zip › suppl_data/Fig.S1_R1.pdf]

Fig. S2

A.

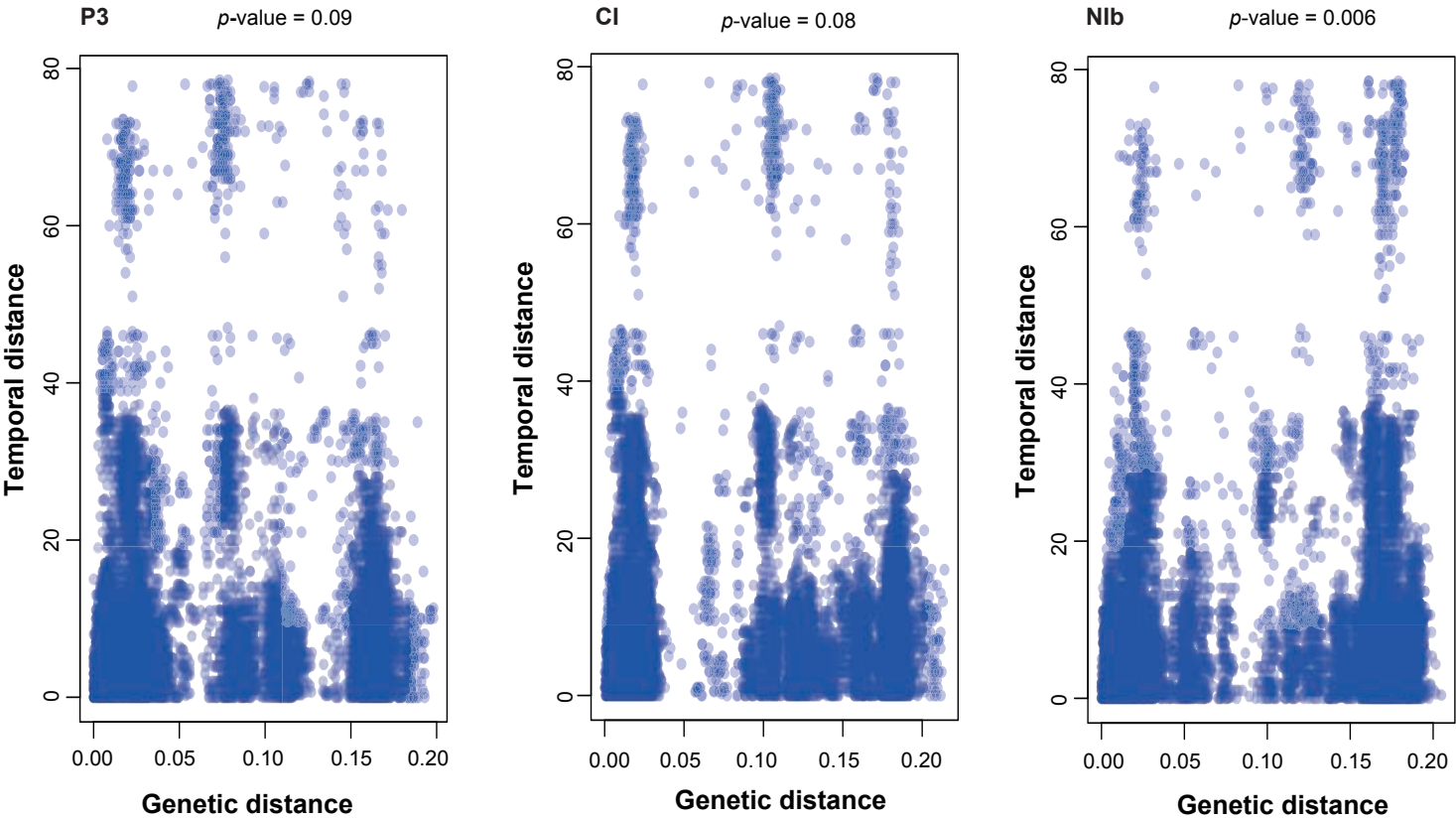

B.

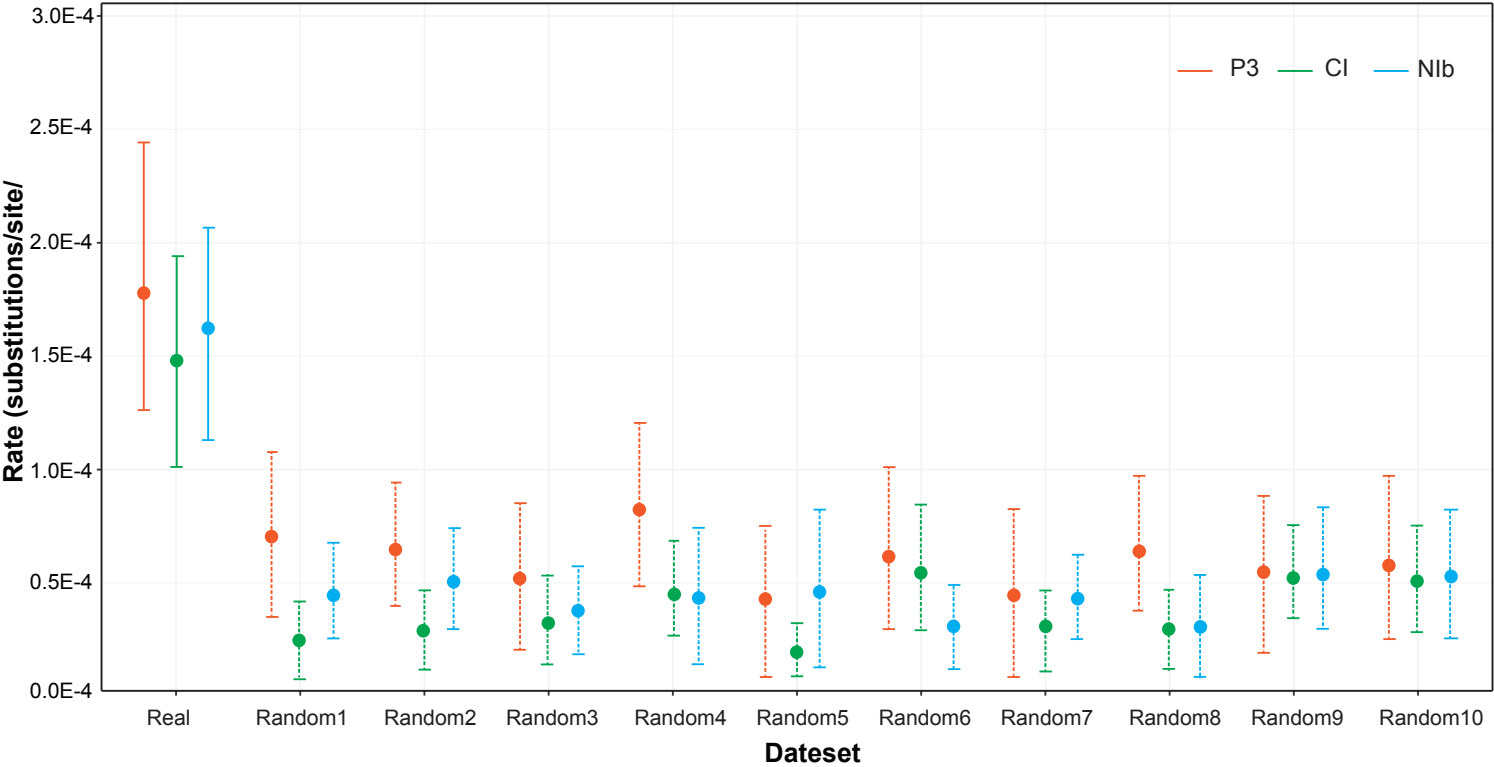

Supplement: veaa056_Supplementary_Data [file veaa056_supplementary_data.zip › suppl_data/Fig.S2_R1.pdf]

Fig. S3

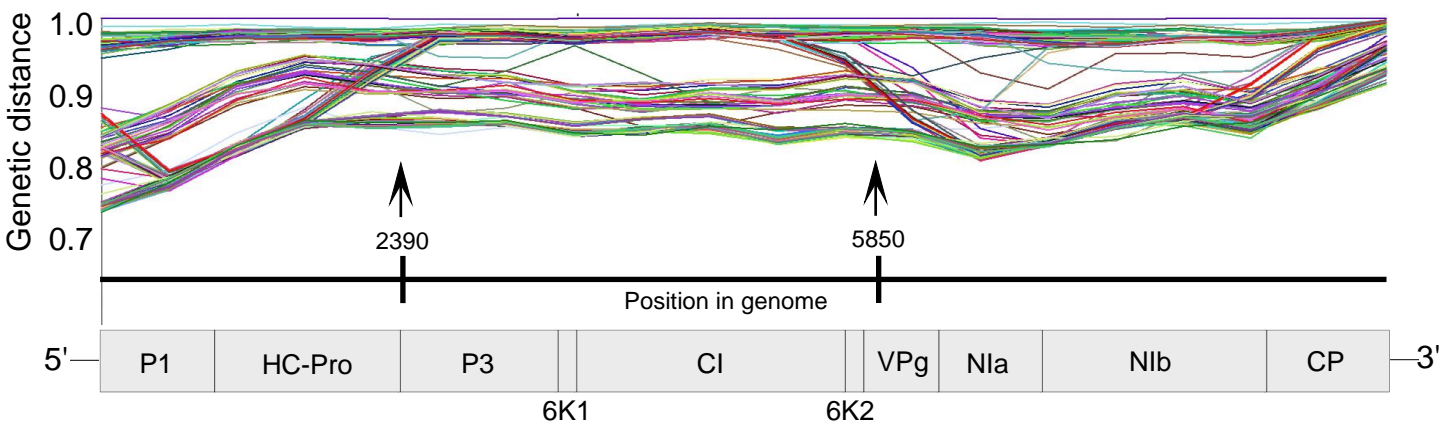

Supplement: veaa056_Supplementary_Data [file veaa056_supplementary_data.zip › suppl_data/Fig.S3_R1.pdf]

Fig. S4

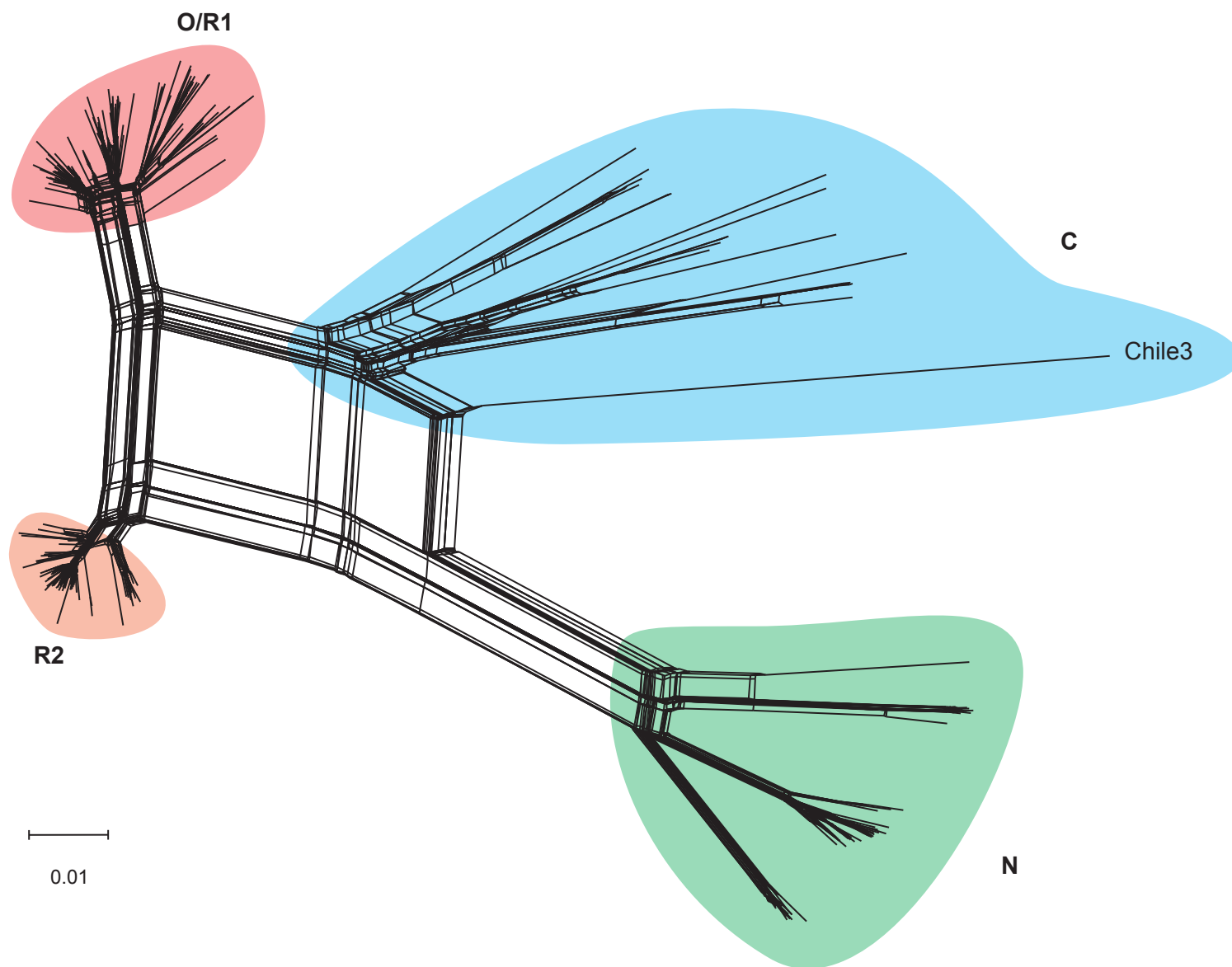

Supplement: veaa056_Supplementary_Data [file veaa056_supplementary_data.zip › suppl_data/Fig.S4_R1.pdf]

Fig. S5

Dataset and genomic region

A.

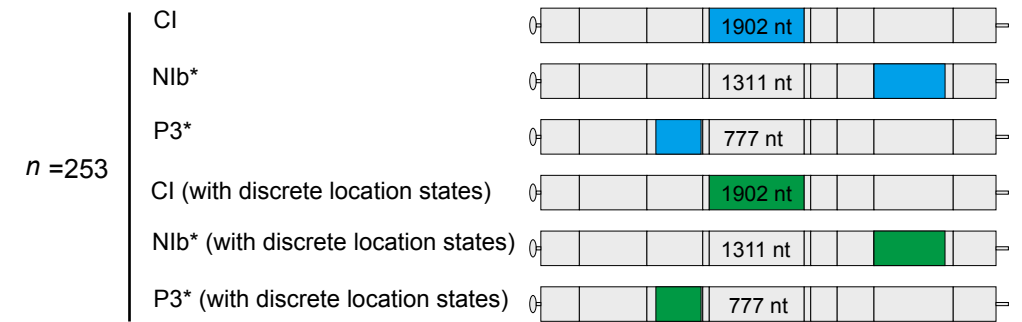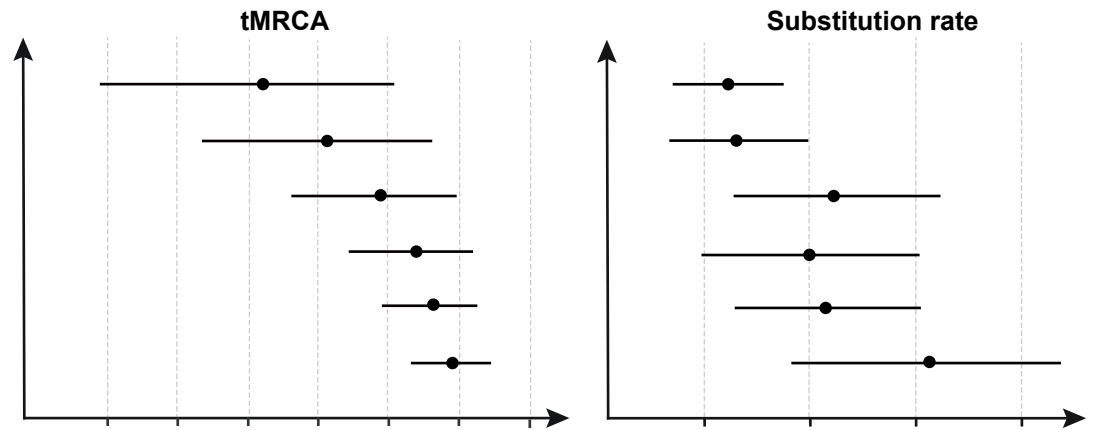

B.

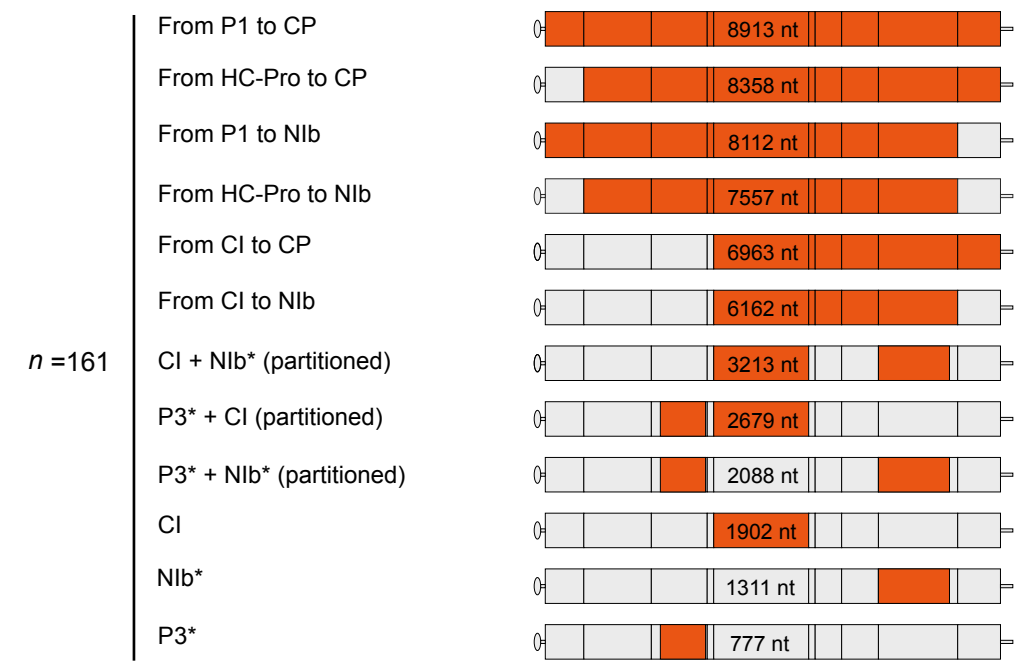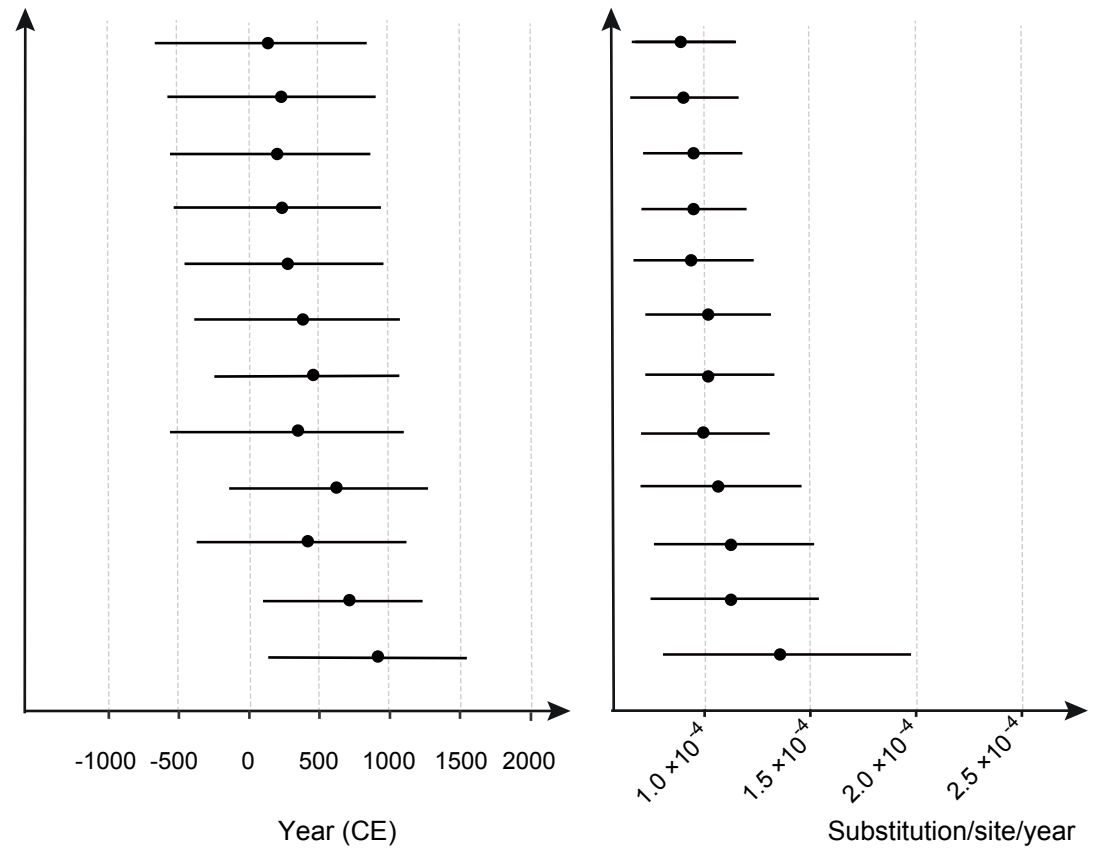

Supplement: veaa056_Supplementary_Data [file veaa056_supplementary_data.zip › suppl_data/Fig.S5_R1.pdf]

Fig. S6

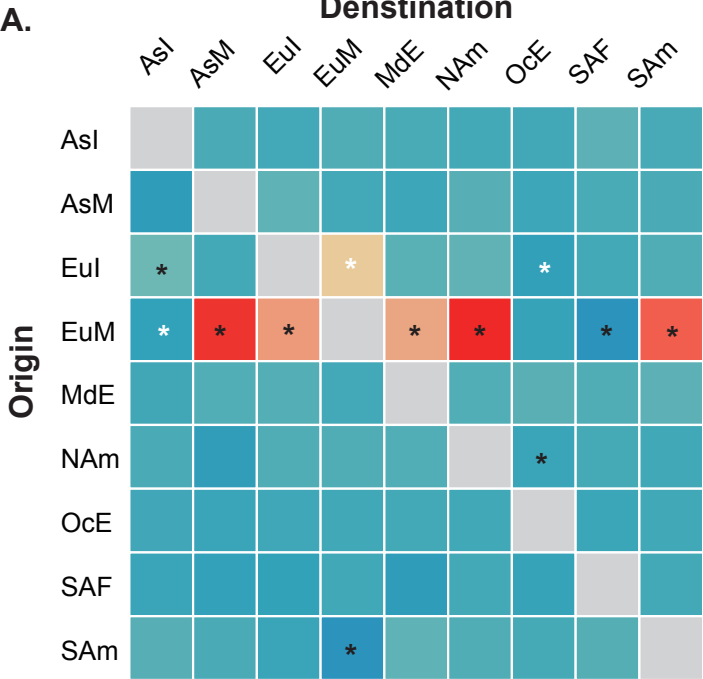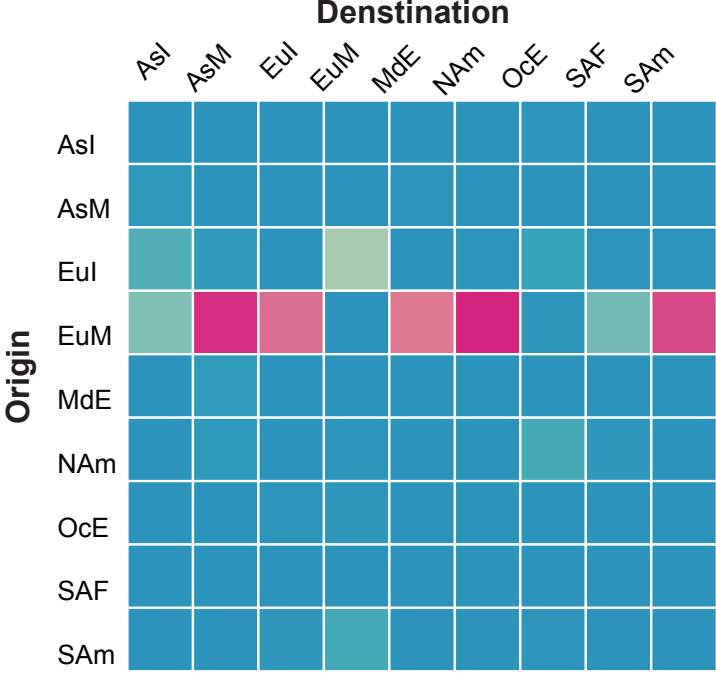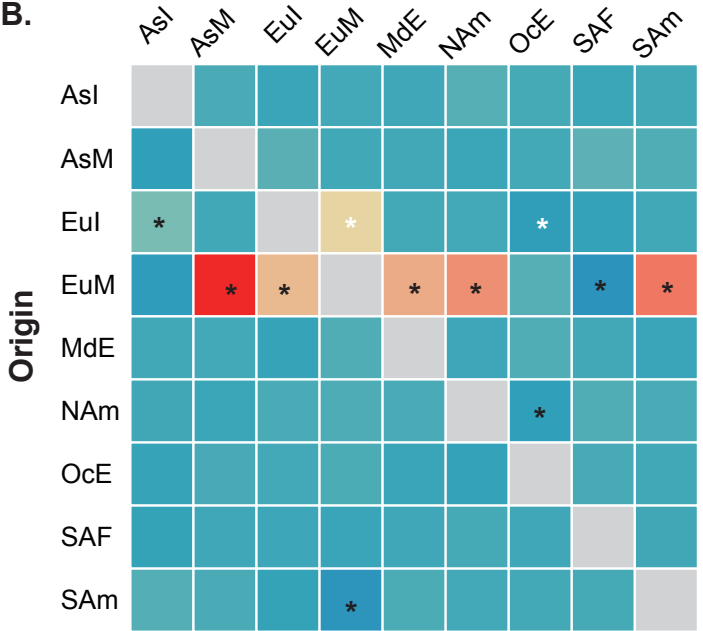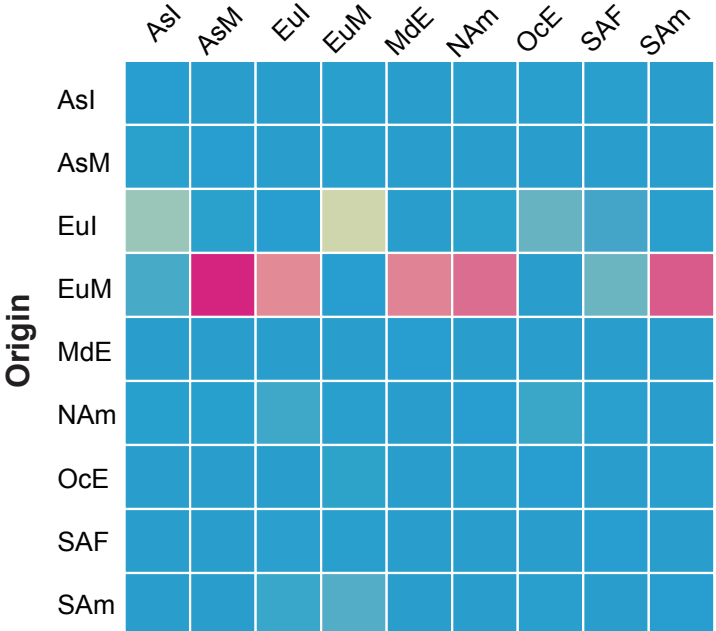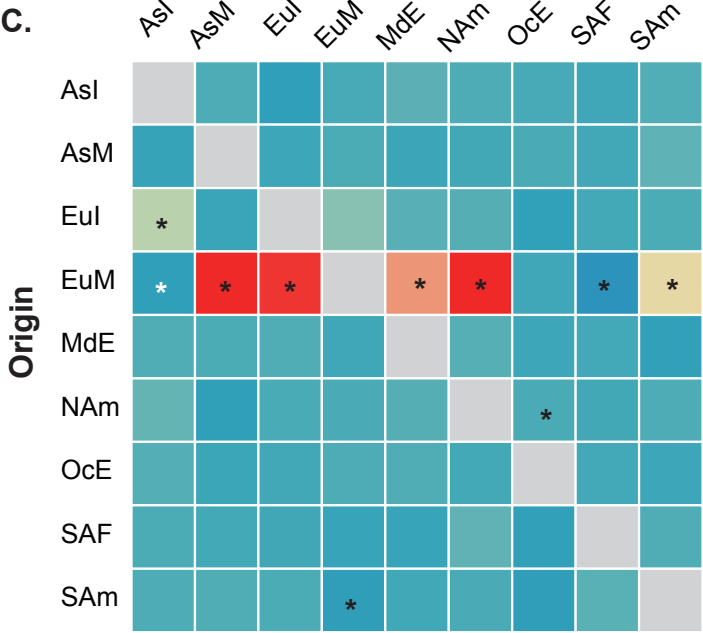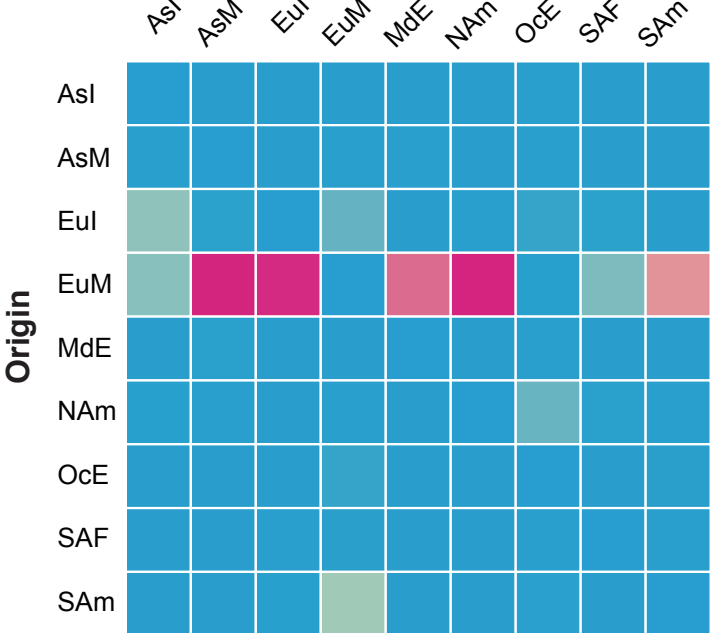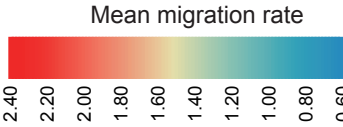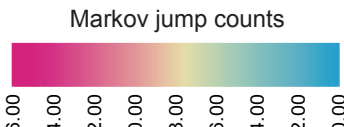

Supplement: veaa056_Supplementary_Data [file veaa056_supplementary_data.zip › suppl_data/Fig.S6_R1.pdf]
